# Cerebral blood flow asymmetry in motor-related regions is associated with 30-day upper-limb motor recovery after acute ischemic stroke

**DOI:** 10.3389/fnins.2026.1955245

**Published:** 2026-10-30

**Authors:** Yongwei Shi, Yichen Lv, Shaoge Gao, Cancan Bi, Fangju Hu, Xi Zhu, Hongyu Zhou, Xin Wang

**Affiliations:** 1 Department of Neurology, Taizhou Fourth People’s Hospital, Taizhou, Jiangsu, China; 2 Department of Rehabilitation Medicine, Northern Jiangsu People’s Hospital, Yangzhou, Jiangsu, China; 3 Department of Radiology, Northern Jiangsu People’s Hospital, Yangzhou, Jiangsu, China

**Keywords:** acute ischemic stroke, arterial spin labeling, asymmetry index, cerebral blood flow, motor recovery, prognosis

## Abstract

**Background:**

Upper-limb motor impairment is a leading cause of long-term disability after acute ischemic stroke (AIS), yet early prognostication relies mainly on clinical scales. Cerebral blood flow (CBF) asymmetry measured by arterial spin labeling (ASL) perfusion MRI may indicate recovery potential. We investigated whether acute-phase CBF asymmetry indices (AIs) in six motor-related regions are associated with 30-day upper-limb motor recovery.

**Methods:**

Sixty patients with AIS underwent pseudo-continuous ASL MRI within 72 h of onset and completed the 30-day Fugl-Meyer Assessment of the Upper Extremity (FMA-UE). Patients were classified into good (*n* = 30, ΔFMA-UE ≥ 10) and poor (*n* = 30, ΔFMA-UE ≤ 4) recovery groups; both thresholds lie outside the published minimal clinically important difference range, and no patient fell between them. Mean CBF was extracted bilaterally from the precentral and postcentral gyri, supplementary motor area, putamen, pallidum, and thalamus, and AIs were calculated. Regional AI–ΔFMA-UE associations were assessed with Spearman correlation, partial correlation, analysis of covariance, and linear regression.

**Results:**

Precentral and postcentral gyrus AIs each correlated positively with ΔFMA-UE (both Spearman *r* = 0.30, *p* = 0.020; false-discovery-rate *q* = 0.020), persisting after adjustment for cognitive status, baseline FMA-UE, and lesion volume (partial r up to 0.326; all *p* < 0.05); other regional AIs showed negligible correlations (|*r*| < 0.09). In analysis of covariance (follow-up FMA-UE as outcome, baseline FMA-UE as covariate), precentral gyrus AI remained associated with the outcome (β = 0.13, 95% CI 0.01–0.25, *p* = 0.037; after cognitive adjustment, *p* = 0.045). A model combining the six AIs with age and cognitive status explained 29.7% of variance in ΔFMA-UE (*F*(8,51) = 2.69, *p* = 0.015), but adding the AIs to clinical variables alone did not significantly improve explanatory performance (ΔR^2^ = 0.048, *p* = 0.170); this R^2^ reflects the combined imaging–clinical model rather than ASL alone.

**Conclusion:**

Sensorimotor-cortex CBF asymmetry within 72 h of onset was associated with 30-day upper-limb motor recovery after AIS, robust to adjustment for clinical variables. Acute-phase ASL may serve as a noninvasive imaging indicator for recovery-oriented rehabilitation stratification, a hypothesis requiring further validation.

## 1 Introduction

Acute ischemic stroke (AIS) is a leading cause of adult disability worldwide, and more than two-thirds of survivors experience varying degrees of functional impairment requiring long-term rehabilitation (Golubnitschaja et al., 2024). Among these impairments, upper-limb motor dysfunction has a particularly persistent effect on independence in daily life and is a major determinant of long-term disability. Accordingly, clinical guidelines identify restoration of upper-limb function as a core goal of stroke rehabilitation (Winstein et al., 2016). Even when baseline deficit severity, lesion volume, and anatomical location are comparable, recovery trajectories vary considerably across individuals: some patients achieve nearly complete functional restoration, whereas others remain severely dependent (Byblow et al., 2015). This heterogeneity indicates that initial deficit severity alone cannot fully explain recovery potential. Clinicians therefore urgently need tools that can predict recovery trajectories during the acute phase and identify patients most likely to benefit from intensive motor rehabilitation. Such tools would directly inform the allocation of rehabilitation resources (Boyd et al., 2017; Stinear et al., 2012).

Current clinical prognostic assessment relies mainly on clinical scales such as the National Institutes of Health Stroke Scale (NIHSS). These scales quantify the severity of neurological deficits but do not directly characterize recovery potential or guide the prioritization of specific rehabilitation programs (Kerleroux et al., 2021; Tahmi et al., 2022). Blood-based biomarkers are easy to obtain but lack the spatial resolution needed to localize salvageable neural networks (Benemerito et al., 2022). Imaging biomarkers have therefore become a major focus of stroke recovery research. The Stroke Recovery and Rehabilitation Roundtable (SRRR) consensus explicitly recommends incorporating imaging biomarkers into the standard framework of recovery prediction research (Boyd et al., 2017). Studies of corticospinal tract lesion load have shown that structural damage burden is significantly associated with motor outcomes (Feng et al., 2015). The PREP algorithm and its simplified version, PREP2, proposed by Stinear et al., combine clinical assessment with neurophysiological and imaging markers to predict 3-month upper-limb functional outcomes within days of onset, with a validated accuracy of approximately 75% (Stinear et al., 2012; Stinear et al., 2017). However, these frameworks primarily characterize structural integrity, a relatively static dimension, and make limited use of cerebral hemodynamics as a functional marker during the acute phase (Jiang et al., 2021).

Perfusion imaging may help close this gap. In acute-phase clinical decision-making, perfusion imaging has been used to delineate the salvageable ischemic penumbra and identify patients likely to benefit from endovascular treatment (Dhillon et al., 2022). Follow-up studies have also shown that dynamic changes in cerebral blood flow (CBF) can serve as objective indicators of rehabilitation response, and relative CBF thresholds have been proposed for prognostic assessment (Lakhani et al., 2024; Steiner et al., 2021). Arterial spin labeling (ASL) uses arterial water protons as an endogenous tracer and requires no exogenous contrast agent. It is safe for patients with impaired renal function, takes less than 5 min to acquire, and can be incorporated into routine stroke MRI protocols; international consensus recommendations have addressed its technical specifications (Alsop et al., 2015; Iutaka et al., 2023; Li and Wang, 2023). Unlike structural imaging, ASL may capture functional hemodynamic changes that precede anatomical recovery and therefore provide early prognostic information (Li and Wang, 2023). However, compared with structural measures such as lesion volume and corticospinal tract integrity, the evidence supporting perfusion parameters for stroke prognostic assessment remains relatively limited (Schellinger et al., 2010). Systematic studies using standardized acquisition and prespecified analysis frameworks are still lacking.

Among perfusion metrics, interhemispheric asymmetry has shown distinct prognostic potential. In studies of crossed cerebellar diaschisis (CCD), quantification schemes based on a bilateral perfusion asymmetry index (AI) have been widely adopted and have been associated with the severity of neurological deficits and functional outcomes (Komaba et al., 2004; Wang et al., 2019). The multicenter prospective iCAS cohort further found that patients with higher quantitative CBF in the contralesional hemisphere during the acute phase had significantly better 90-day functional outcomes (OR = 4.6, 95% CI: 1.4–14.7), suggesting that contralesional perfusion itself may carry prognostic information (Thamm et al., 2019). Previous studies, however, have largely focused on the relationship between infarct core or hypoperfusion volume and prognosis or have used voxel-level analyses; few have systematically assessed motor-system regions of interest (ROIs). Moreover, existing prognostic frameworks have focused almost exclusively on motor execution structures, with limited attention to the role of sensorimotor integration in recovery. Somatosensory feedback is a necessary input for motor learning and revision of motor plans, and the perfusion status of the sensory cortex may likewise carry information about recovery potential (Edwards et al., 2019; Meyer et al., 2014). Accordingly, this study enrolled patients with AIS and upper-limb motor impairment, extracted CBF from six bilateral motor-related regions (precentral gyrus, postcentral gyrus, supplementary motor area, putamen, pallidum, and thalamus) using the automated anatomical labeling (AAL) atlas, and calculated asymmetry indices. The study aimed to (i) identify region-specific perfusion asymmetry patterns associated with 30-day upper-limb motor recovery after stroke and (ii) evaluate the explanatory power of multi-region AIs combined with clinical variables for motor recovery, thereby providing an imaging basis for individualized rehabilitation stratification.

## 2 Materials and methods

### 2.1 Study design and participants

This was a single-center prospective cohort study that consecutively screened patients with AIS admitted to the Department of Neurology, Northern Jiangsu People’s Hospital, between June 2024 and June 2025. Of 286 patients screened, 177 were excluded according to the inclusion and exclusion criteria and 26 had incomplete baseline data collection; 83 patients were enrolled with a complete baseline assessment and ASL MRI, of whom 17 were lost to the 30-day follow-up (mainly voluntary discharge or clinical worsening) and 6 were excluded for insufficient imaging quality, leaving 60 patients in the final analysis (Figure 1). The study protocol followed the Declaration of Helsinki and was registered with the Chinese Clinical Trial Registry (registration No. ChiCTR2100042325). All participants or their legally authorized representatives provided written informed consent.

**Figure 1:**
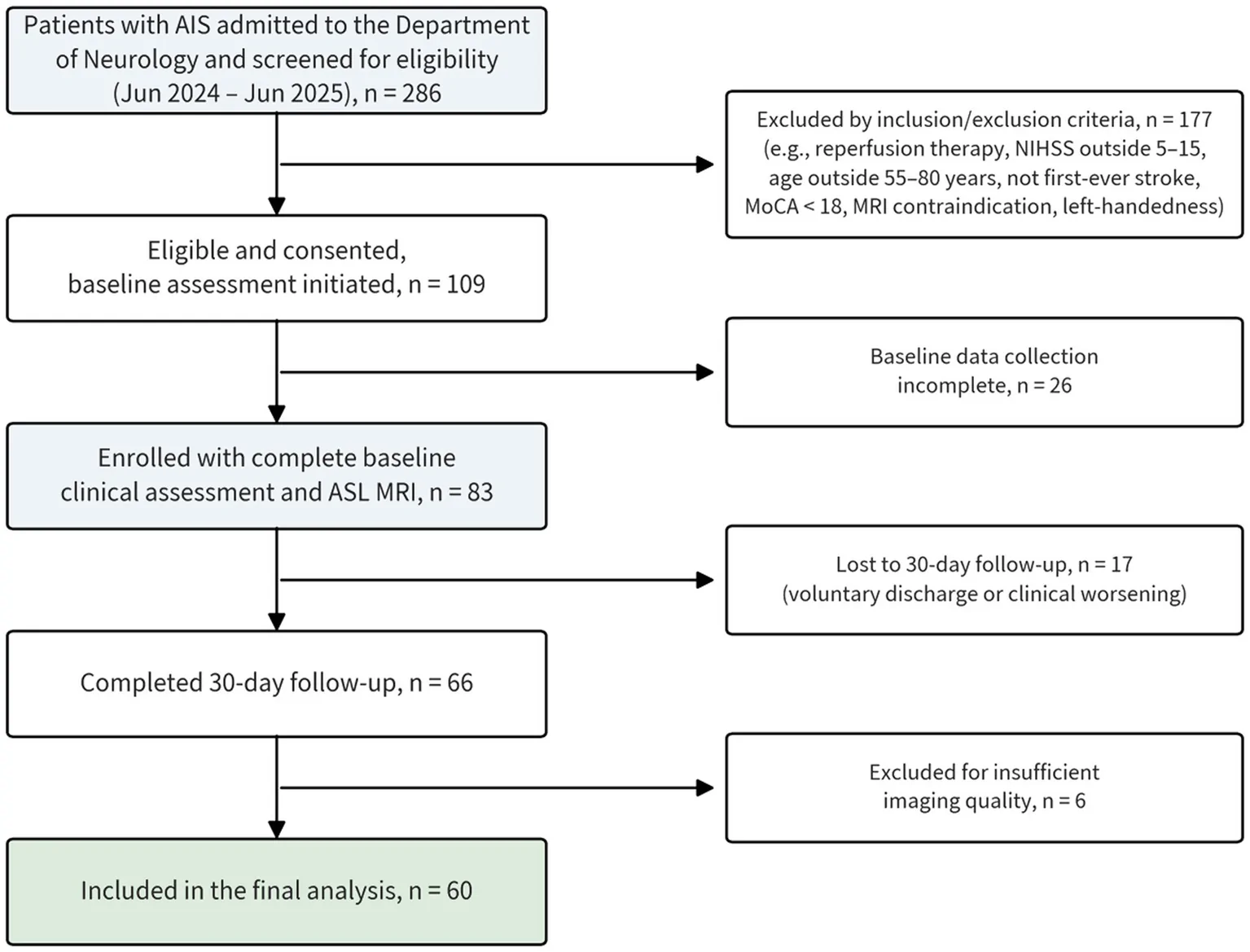
Patient flow diagram. Of 286 patients with acute ischemic stroke screened between June 2024 and June 2025, 177 were excluded according to the inclusion and exclusion criteria, 26 had incomplete baseline data collection, 17 were lost to the 30-day follow-up (mainly voluntary discharge or clinical worsening), and 6 were excluded for insufficient imaging quality, leaving 60 patients in the final analysis.

Inclusion criteria: (i) first-ever AIS meeting the diagnostic criteria of the 2023 Chinese guidelines for the clinical management of cerebrovascular diseases; (ii) MRI completed within 72 h of onset; (iii) transfer to the rehabilitation medicine department on the 8th to 10th day after stroke onset; (iv) no intravenous thrombolysis or mechanical thrombectomy; (v) baseline NIHSS score of 5–15; (vi) aged 55–80 years; (vii) Montreal Cognitive Assessment (MoCA) score ≥ 18; and (viii) right-handedness.

Exclusion criteria: (i) hemorrhagic stroke; (ii) severe hepatic or renal failure; (iii) contraindications to MRI; and (iv) a history of neurological or psychiatric disease.

Sample size considerations: the primary analysis was the correlation between regional CBF asymmetry indices and ΔFMA-UE. With 60 participants, the study provides 80% power at a two-sided α of 0.05 to detect correlations of *r* ≥ 0.35, and approximately 65% power to detect a correlation of the magnitude observed for the sensorimotor cortical AIs (*r* = 0.30). Analyses of effects smaller than *r* ≈ 0.35 are underpowered and are therefore reported as exploratory.

### 2.2 Clinical protocol

Baseline MRI was completed within 72 h of stroke onset, and functional outcome was assessed at the 30-day follow-up. Trained neurologists recorded NIHSS scores, and multiparametric 3.0T MRI, including diffusion-weighted imaging (DWI) and ASL, was performed. On the 8th to 10th day after stroke onset, patients were transferred to the rehabilitation department and received standard care, including blood pressure and lipid management, physical therapy, and occupational therapy, without experimental adjunctive treatments. At 30 days, the FMA-UE was administered by two certified neurologists blinded to the imaging data, and the cohort was divided into good and poor recovery groups according to ΔFMA-UE.

### 2.3 Clinical data collection

Baseline stroke severity was quantified with the NIHSS (total score 0–42; higher scores indicate more severe neurological deficits). Upper-limb motor function was assessed with the FMA-UE (range 0–66; higher scores indicate better motor function). The FMA-UE was repeated at the 30-day follow-up, and the change was calculated as ΔFMA-UE = follow-up FMA-UE − baseline FMA-UE. For descriptive group comparisons, patients with ΔFMA-UE ≥ 10 points were assigned to the good recovery group and those with ΔFMA-UE ≤ 4 points to the poor recovery group. Both thresholds lie outside the published estimates of the minimal clinically important difference (MCID) for the FMA-UE: 4.25–7.25 points in chronic stroke (Page et al., 2012) and 9–13 points in the acute-to-subacute phase, the phase examined in the present study (Huynh et al., 2023; for a review, see Mishra et al., 2024), so the two groups represent clinically unambiguous recovery states rather than an arbitrary median split. No enrolled patient had a ΔFMA-UE between 5 and 9 points; consequently, no participant was excluded by this dichotomization, and the balanced group sizes (30/30) reflect 60 consecutively enrolled patients without any outcome-based selection or matching. A sensitivity analysis using ΔFMA-UE ≥ 5 as the cut-off yielded an identical grouping and a consistent pattern of results (Supplementary Table S2). All primary inferences are based on continuous-variable analyses; the dichotomization is used for descriptive purposes only. MoCA score, age, sex, lesion side, lesion volume, and cortical involvement were also recorded.

### 2.4 MRI data acquisition

Participants lay supine with their eyes closed and were instructed to refrain from thinking of anything in particular. Foam pads were used to minimize head motion, and no participant was excluded for excessive motion (threshold 2 mm/2°). Imaging was performed on a 3.0 T GE Discovery MR750 scanner equipped with a 32-channel head coil.

(1) High-resolution T1-weighted structural imaging (three-dimensional magnetization-prepared rapid gradient echo, 3D MPRAGE): TR 2300 ms; TE 2.98 ms; inversion time (TI) 900 ms; flip angle 9°; field of view (FOV) 240 × 240 mm^2^; matrix 256 × 256; 172 sagittal slices; slice thickness 1 mm; no gap. A T2-weighted fluid-attenuated inversion recovery (T2-FLAIR) sequence was acquired for lesion visualization: TR 9000 ms; TE 120 ms; TI 2500 ms; slice thickness 5 mm; no gap.
(2) Three-dimensional pseudo-continuous ASL (3D pCASL) with background suppression and a single post-labeling delay (PLD) of 2050 ms: TR 4836 ms; TE 10.3 ms; flip angle 111°; FOV 220 × 220 mm^2^; matrix 128 × 128; 35 axial slices; slice thickness 4.5 mm; no gap; label duration 1,500 ms; 8 spiral arms with 512 points each; total acquisition time 4 min 12 s. CBF maps were generated automatically using the continuous gradient (CG) method in the vendor’s FuncTool software (version 4.6, GE Healthcare), which implements a single-compartment quantification model consistent with the ISMRM/ESMRMB ASL consensus recommendations (Alsop et al., 2015); quantification used the vendor’s default parameters, no separate M0 image was acquired, and no partial-volume correction was applied.

Acute infarction was confirmed on diffusion-weighted imaging (DWI) in all participants, and the interval from stroke onset to MRI was within 72 h in every case (inclusion criterion ii). The single PLD of 2,050 ms was selected in accordance with the ISMRM/ESMRMB consensus recommendation of a PLD of at least 2,000 ms for populations in whom delayed arterial transit is expected, such as patients with cerebrovascular disease (Alsop et al., 2015). Vascular imaging (MRA or CTA) was not performed as part of the study protocol; however, patients with suspected large-vessel occlusion are routinely referred for reperfusion therapy at our center and were therefore largely excluded from this cohort by the eligibility criteria (no intravenous thrombolysis or mechanical thrombectomy; NIHSS 5–15), so that severe proximal arterial occlusions are unlikely to be prevalent in the analyzed sample. The potential influence of residual arterial transit time effects is addressed in Section 4.6.

### 2.5 Image preprocessing

All analyses were performed in MATLAB R2020b (SPM12). ASL images were realigned to the first volume, and participants with displacement exceeding 2 mm/2° were excluded. CBF maps were coregistered to individual T1 images, normalized to MNI space, resampled to 2 × 2 × 2 mm^3^, and smoothed with an 8 mm FWHM Gaussian kernel. All ROI-level CBF values and asymmetry indices were computed from these quantitative CBF maps (mL/100 g/min); no z-score transformation was applied at any stage of the ROI-level analysis. Images were not flipped; all analyses were performed in the native MNI orientation, and ipsilesional and contralesional ROIs were defined individually for each patient according to the lesion side confirmed on DWI and the lesion mask.

Regions of interest were defined with the original automated anatomical labeling (AAL) atlas (Tzourio-Mazoyer et al., 2002), and mean CBF values were extracted from the following bilateral motor-related regions: precentral gyrus, postcentral gyrus, supplementary motor area (SMA), putamen, pallidum, and thalamus. Ipsilesional and contralesional CBF values were recorded for each ROI. Infarcted tissue within an ROI was not excluded from the CBF measurement; ROI means therefore reflect the average perfusion of the entire anatomical region, including any infarcted portions, and lesion volume was carried into the adjusted analyses to account for this (Sections 3.3 and 3.4).

The analysis focused on the bilateral precentral and postcentral gyri. This choice was based on the following anatomical and technical considerations: first, the precentral and postcentral gyri correspond to the primary motor cortex and the primary somatosensory cortex, respectively, and are key cortical nodes of the upper-limb sensorimotor circuit that are most directly related to motor recovery after stroke (Edwards et al., 2019; Meyer et al., 2014); second, with single-PLD ASL acquisition, the signal-to-noise ratio of cortical gray matter is higher than that of deep gray matter structures, and deep nuclei are more susceptible to lesion occupancy, so cortical ROI-based perfusion asymmetry measurements have better reproducibility (Alsop et al., 2015); and third, the role of sensorimotor integration in motor learning (Zandvliet et al., 2020) suggests that sensorimotor cortices are more likely than deep structures to carry recovery-related information. For multiplicity control, these two sensorimotor cortical ROIs were designated *a priori* as the primary testing family (Section 2.7); the remaining four regions (SMA, putamen, pallidum, and thalamus) were analyzed exploratorily and are reported as observed (see Section 3.3) for reference in future studies.

### 2.6 Calculation of the CBF asymmetry index

The CBF asymmetry index (AI) was calculated with reference to previous perfusion imaging studies: AI = (CBF_ipsilesional − CBF_contralesional) / (CBF_ipsilesional + CBF_contralesional) × 100%. AI > 0 indicates higher ipsilesional than contralesional perfusion; AI < 0 indicates lower ipsilesional than contralesional perfusion; and a larger absolute AI indicates more pronounced bilateral perfusion asymmetry. Because the index is computed from quantitative CBF values, the denominator (CBF_ipsilesional + CBF_contralesional) is a sum of two non-negative perfusion values and cannot approach zero under physiological conditions; the index therefore retains its interpretation as a normalized perfusion ratio. Because the AI is a signed relative measure, a positive correlation between AI and recovery in a cohort in which mean AIs are negative indicates that AI values closer to zero—i.e., more symmetric bilateral perfusion—are associated with better recovery; the index by itself cannot distinguish whether such symmetry reflects preserved ipsilesional perfusion, increased contralesional perfusion, or both.

### 2.7 Statistical analysis

Statistical analyses were performed in SPSS 25.0 (IBM Corp., Armonk, NY, USA) and MATLAB R2020b (SPM12). Baseline characteristics (Table 1) were tested for normality and homogeneity of variance with the Shapiro–Wilk and Levene tests, between-group comparisons of continuous variables were performed with Welch’s t test, and categorical variables with the χ^2^ test; missing data were handled by listwise deletion.

**Table 1:** Baseline clinical characteristics of the two groups.

Characteristic	Overall (*n* = 60)	Good recovery (*n* = 30)	Poor recovery (*n* = 30)	*p* value
Age	65.68 ± 6.76	64.57 ± 6.83	66.80 ± 6.62	0.204
Baseline FMA-UE	25.33 ± 5.70	25.53 ± 5.46	25.13 ± 6.01	0.788
Follow-up FMA-UE	32.37 ± 7.70	37.70 ± 5.18	27.03 ± 5.92	<0.001
ΔFMA-UE	7.03 ± 5.37	12.17 ± 1.64	1.90 ± 1.21	<0.001
MoCA	24.85 ± 3.00	26.30 ± 2.29	23.40 ± 2.94	<0.001
Lesion volume (voxels)	4982.22 ± 7166.22	2418.87 ± 4731.74	7545.57 ± 8276.24	0.005
Lesion volume (ml)	39.86 ± 57.33	19.35 ± 37.85	60.36 ± 66.21	0.005
Sex (male/female)	36/24	20/10	16/14	0.429
Lesion side (left/right)	29/31	13/17	16/14	0.605
Cortical involvement (yes/no)	46/14	23/7	23/7	1.000

Lesion volume (mL) = number of lesion voxels × 8/1,000. Continuous variables were compared between groups with Welch’s t test and categorical variables with the χ^2^ test. MoCA differed between groups (*t* = 4.26, *p* < 0.001); lesion volume differed between groups (*t* = −2.95, *p* = 0.005). FMA-UE, Fugl-Meyer Assessment of the Upper Extremity; MoCA, Montreal Cognitive Assessment.

For the ROI analysis (Table 2), Welch’s t tests were used to compare regional AIs between groups, and Cohen’s d was calculated as the effect size with bootstrap 95% confidence intervals (1,000 resamples). Spearman rank correlation analysis was used to assess the correlations of regional AIs with baseline FMA-UE, follow-up FMA-UE, ΔFMA-UE, age, and MoCA score. Linear regression analyses included (i) univariable linear regression of each ROI AI on ΔFMA-UE and (ii) multivariable linear regression combining the six regional AIs with age and MoCA score for ΔFMA-UE. The significance level was set at a two-sided α = 0.05.

**Table 2:** Comparison of CBF asymmetry indices in motor-related regions between the two groups.

ROI	Good recovery	Poor recovery	Mean diff.	*t*	*p*	Cohen’s d (95% CI)
Precentral gyrus	−4.70 ± 8.30	−9.36 ± 13.64	4.67	1.600	0.116	0.413 (−0.13 to 0.97)
Postcentral gyrus	−4.11 ± 6.88	−7.29 ± 16.40	3.18	0.980	0.333	0.253 (−0.25 to 0.88)
Supplementary motor area	−2.60 ± 5.68	−3.27 ± 7.11	0.67	0.402	0.689	0.104 (−0.43 to 0.57)
Putamen	−4.64 ± 5.32	−4.97 ± 11.97	0.34	0.141	0.889	0.036 (−0.54 to 0.52)
Pallidum	−2.89 ± 7.38	−3.16 ± 10.73	0.27	0.115	0.909	0.030 (−0.50 to 0.53)
Thalamus	−4.98 ± 7.51	−4.06 ± 6.01	−0.92	−0.522	0.603	−0.135 (−0.63 to 0.39)

Values are mean ± SD. AI = (CBF_ipsilesional − CBF_contralesional) / (CBF_ipsilesional + CBF_contralesional) × 100%. Between-group comparisons were performed with Welch’s *t* test. Cohen’s d 95% confidence intervals were estimated by bootstrap (1,000 resamples). ROI, region of interest.

At the study level, no primary or secondary imaging endpoints were designated in the trial registration, and the findings of this observational analysis are therefore reported as exploratory and hypothesis-generating overall. Within the analysis itself, to contain multiplicity risk, the two sensorimotor cortical ROIs with the strongest anatomical priors (precentral and postcentral gyri) were designated *a priori* as the primary testing family, within which Benjamini–Hochberg false discovery rate (FDR) control was applied; the remaining four regions were analyzed exploratorily and are reported with unadjusted *p* values. Because baseline MoCA scores and lesion volume differed between the two groups, partial correlation analysis based on rank-transformed residuals was used to evaluate the robustness of the associations after controlling for MoCA score, baseline FMA-UE, and lesion volume; analysis of covariance (ANCOVA) with follow-up FMA-UE as the outcome and baseline FMA-UE as a covariate was performed to address regression to the mean and proportional-recovery effects.

## 3 Results

### 3.1 Baseline characteristics

Sixty patients with AIS were included, with 30 in the good recovery group (ΔFMA-UE ≥ 10) and 30 in the poor recovery group (ΔFMA-UE ≤ 4). The mean age was 64.57 ± 6.83 years in the good recovery group and 66.80 ± 6.62 years in the poor recovery group (overall range, 55–80 years). As shown in Table 1, the two groups did not differ significantly in age, sex, lesion side, cortical involvement, or baseline FMA-UE (all *p* > 0.05). However, MoCA scores were higher in the good recovery group than in the poor recovery group (26.30 ± 2.29 versus 23.40 ± 2.94, *p* < 0.001), and lesion volume was larger in the poor recovery group (60.36 ± 66.21 versus 19.35 ± 37.85 mL, *p* = 0.005); both variables were therefore carried into adjusted analyses (Sections 3.3 and 3.4).

### 3.2 Comparison of CBF asymmetry indices in motor-related regions

Descriptive statistics and between-group comparisons of CBF AI in motor-related regions are shown in Table 2. Mean AIs were negative in both groups, indicating that ipsilesional perfusion was generally lower than contralesional perfusion. Compared with the poor recovery group, the good recovery group had smaller absolute AIs in the precentral and postcentral gyri, indicating bilateral perfusion closer to symmetry; given the signed and relative nature of the AI, this pattern cannot by itself be attributed to preserved ipsilesional perfusion rather than to contralesional changes. The between-group difference was largest for precentral gyrus AI (good recovery: −4.70 ± 8.30; poor recovery: −9.36 ± 13.64; Cohen’s d = 0.41, small-to-medium effect size), although it was not statistically significant (*p* = 0.116). Between-group differences in the remaining regions were not significant (*p* > 0.05; Table 2 and Figure 2). As shown in Figure 3A, bilateral hemispheric CBF was overall higher in the good recovery group than in the poor recovery group, and the ipsilesional–contralesional perfusion gap was most pronounced in the sensorimotor cortex; Figure 3B shows that the between-group differences in AI at the individual level were consistent and were not driven by a few outliers. The anatomical distribution of the between-group mean CBF differences is shown in Figure 4 (for visualization only).

**Figure 2:**
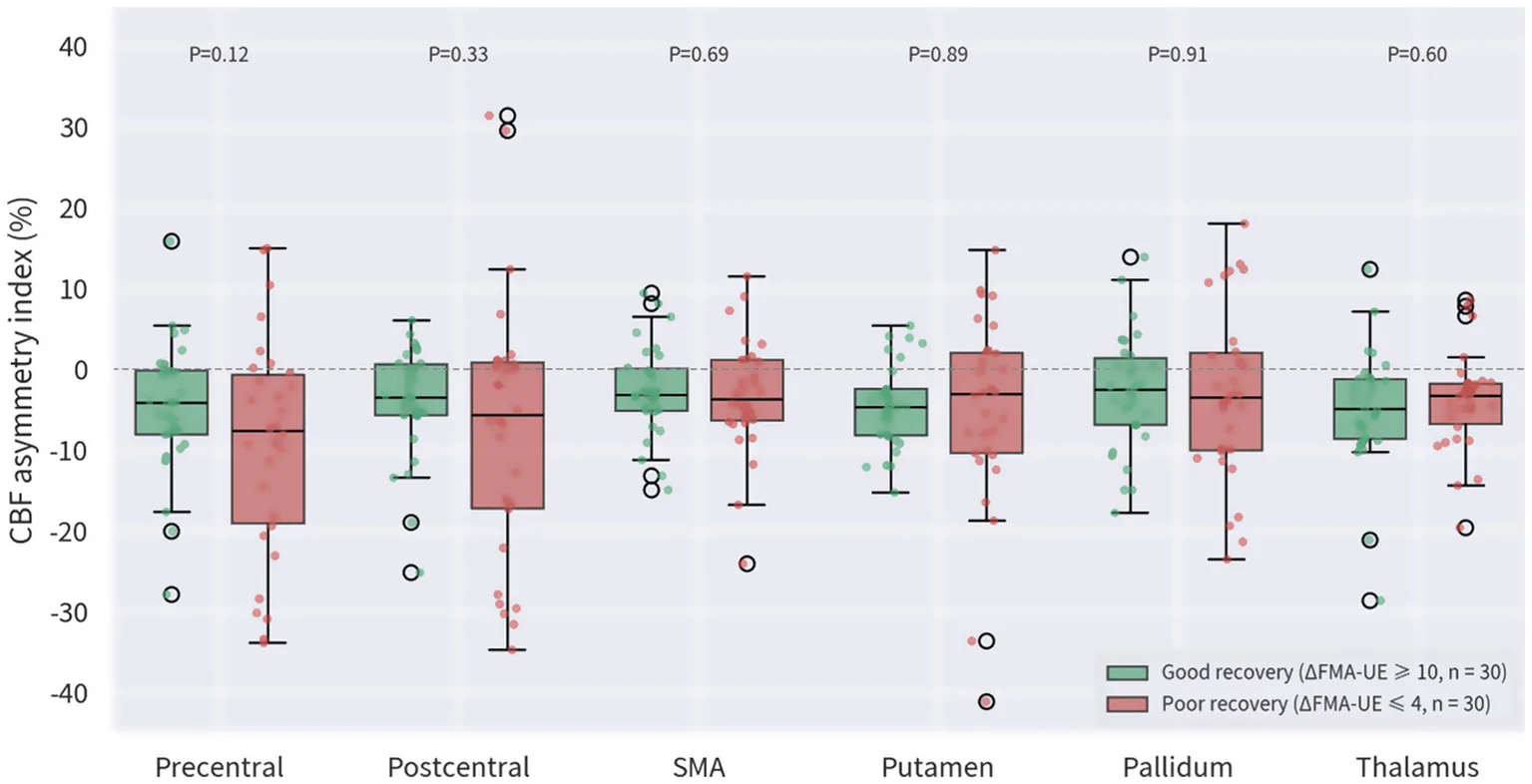
Cerebral blood flow (CBF) asymmetry indices (AIs) by recovery group across six motor-related regions of interest. Boxes show median and interquartile range; whiskers show 1.5 × IQR; dots show individual patients. Between-group comparisons used Welch’s t tests (Table 2).

**Figure 3:**
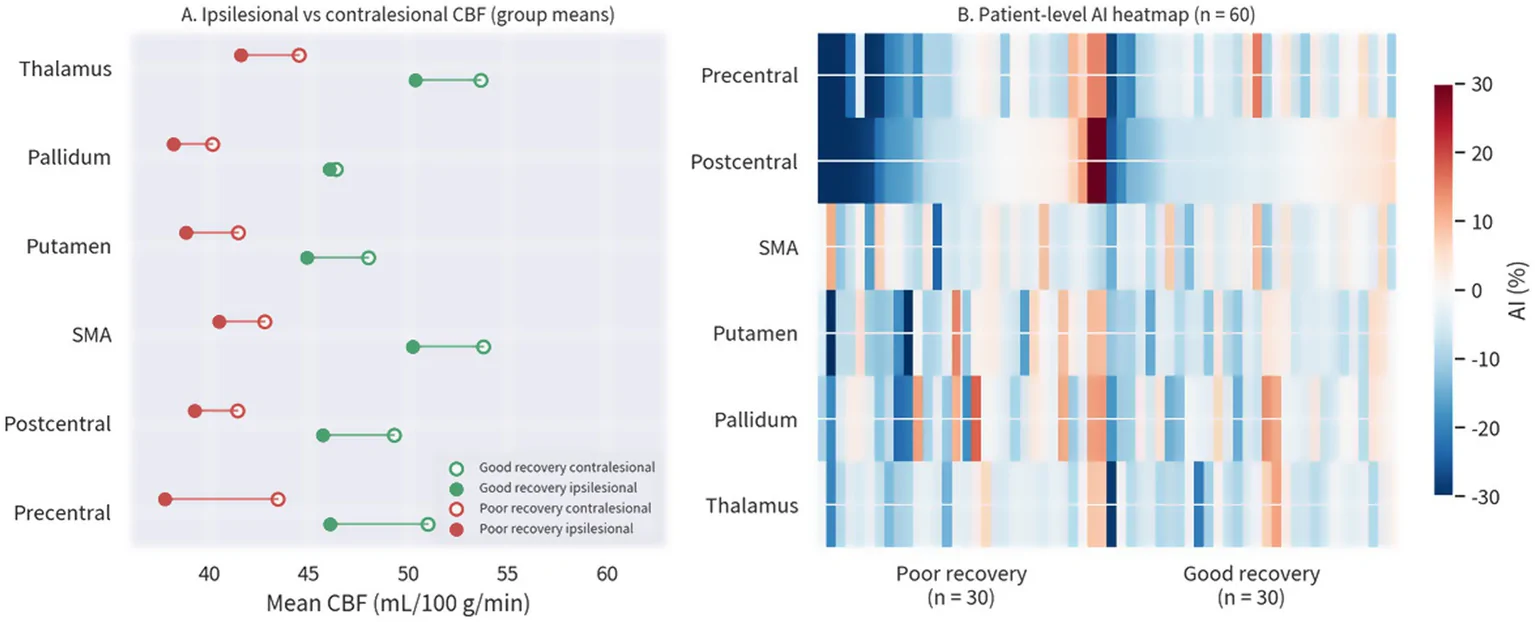
**(A)** Ipsilesional- versus contralesional-hemisphere CBF (group means) for six motor-related regions (dumbbell plot); **(B)** patient-level AI heatmap (*n* = 60, sorted by group and postcentral gyrus AI).

**Figure 4:**
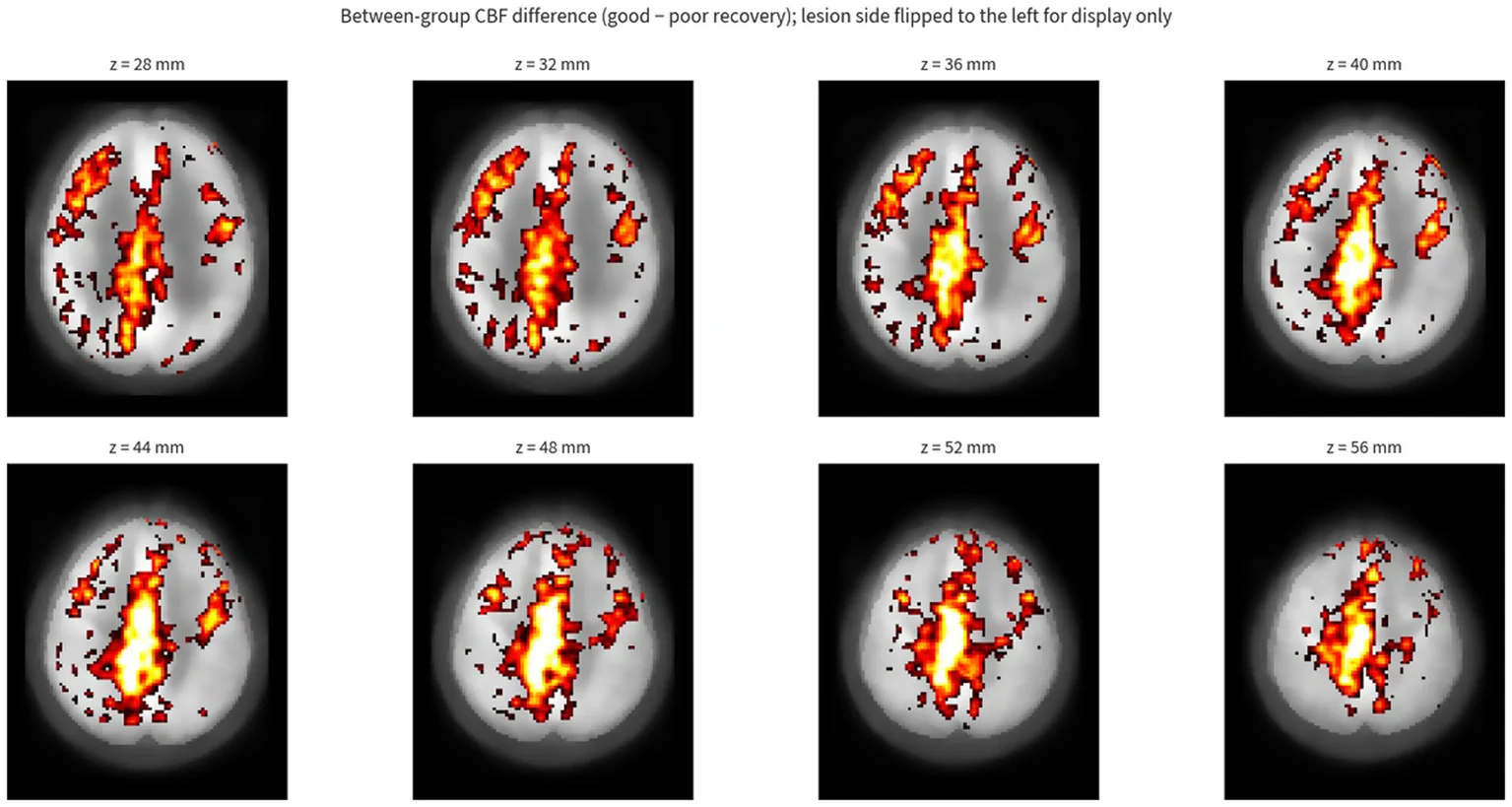
Between-group differences in cerebral blood flow (good minus poor recovery, *n* = 30 per group) overlaid on the mean CBF template (axial slices, MNI z = 28–56 mm). For display purposes only, CBF maps were flipped so that the lesion side appears uniformly on the anatomical left; all ROI-level analyses were performed in native MNI orientation with per-patient ipsilesional/contralesional definitions. Warmer colors indicate higher perfusion in the good-recovery group. Display threshold: 70th percentile of |ΔCBF| within AAL cerebral gray matter; per-subject CBF was clipped at 90 mL/100 g/min to suppress superior-sagittal-sinus signal. For visualization only; statistical inference was performed at the ROI level (Table 2).

### 3.3 Correlations between CBF AI and motor recovery

Spearman correlation analysis showed that precentral gyrus AI (*r* = 0.299, 95% CI 0.02–0.54, *p* = 0.020; Figure 5) and postcentral gyrus AI (*r* = 0.299, 95% CI 0.03–0.54, *p* = 0.020; Figure 6) were each positively correlated with ΔFMA-UE; both associations survived Benjamini–Hochberg FDR control within the prespecified sensorimotor family (both *q* = 0.020). Correlations of the SMA, putamen, pallidum, and thalamus AIs with ΔFMA-UE were negligible (|*r*| < 0.09). In univariable linear regression, precentral gyrus AI explained the largest proportion of variance in ΔFMA-UE (R^2^ = 0.072, *p* = 0.038), followed by postcentral gyrus AI (R^2^ = 0.040, *p* = 0.125). The full Spearman correlation matrix is provided in Table 3.

**Figure 5:**
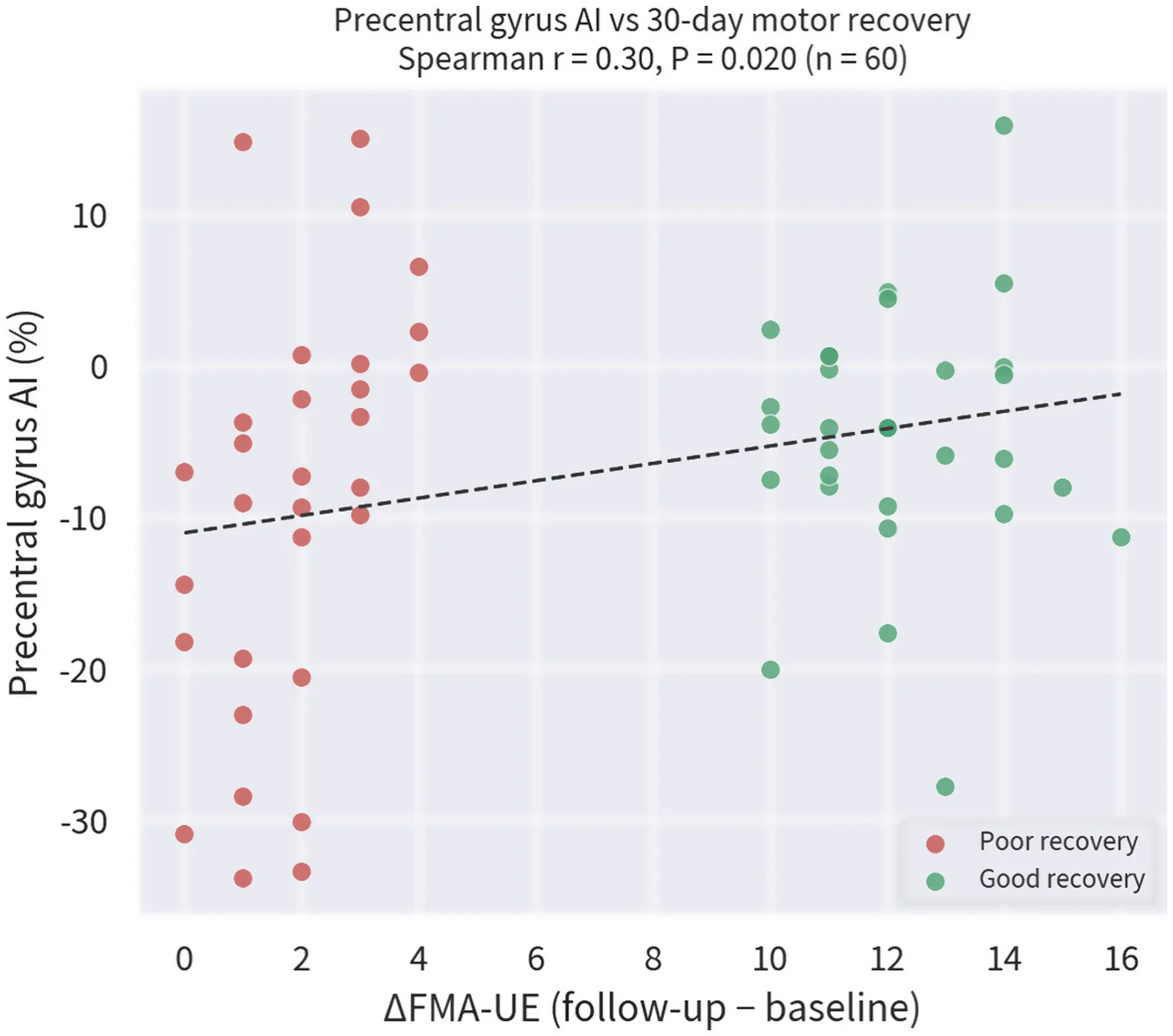
Association between precentral gyrus AI and 30-day motor recovery (ΔFMA-UE) (Spearman *r* = 0.299, 95% CI 0.02–0.54, *p* = 0.020). Each dot represents one patient (*n* = 60); colors denote recovery groups.

**Figure 6:**
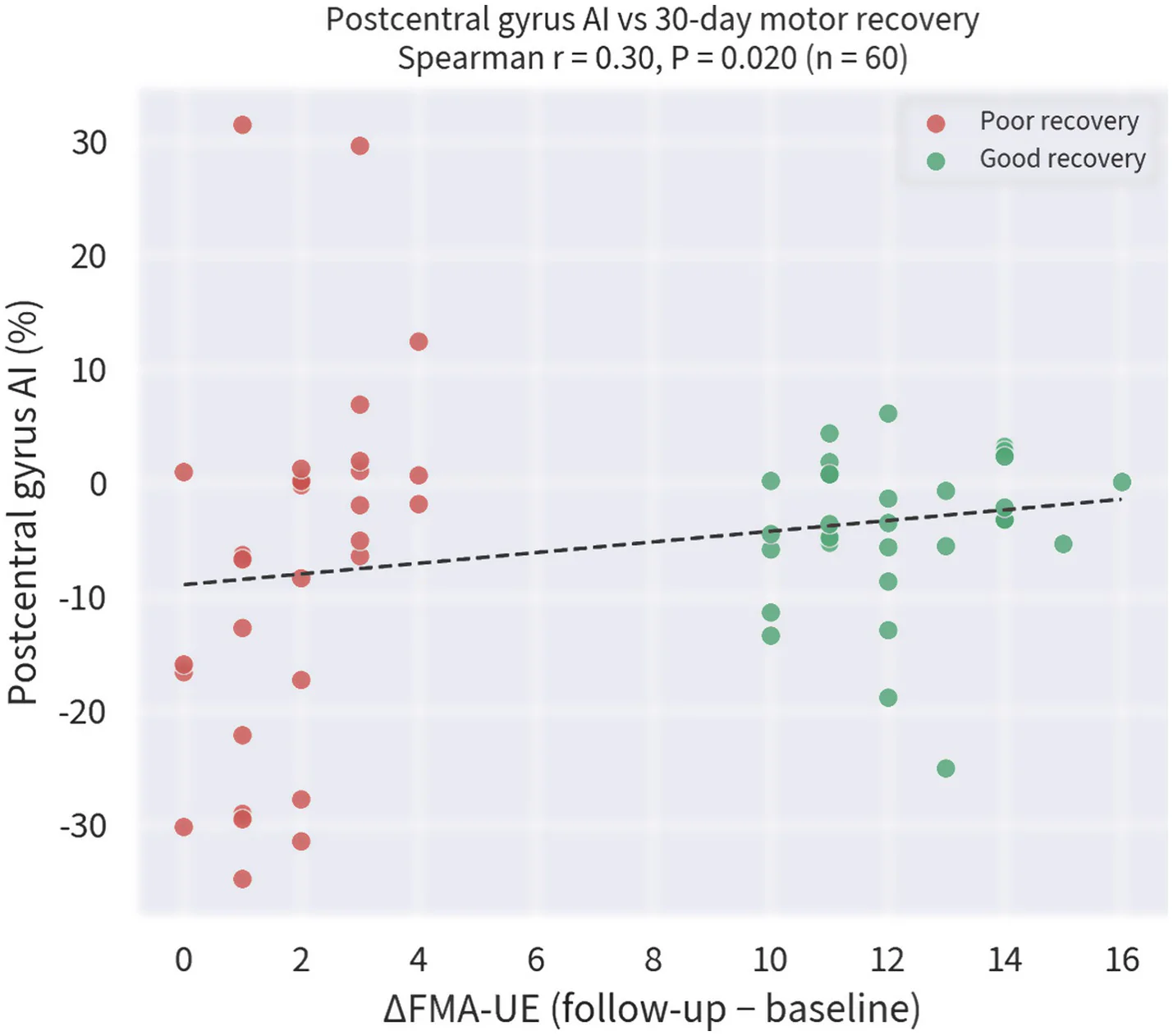
Association between postcentral gyrus AI and 30-day motor recovery (ΔFMA-UE) (Spearman *r* = 0.299, 95% CI 0.03–0.54, *p* = 0.020). Each dot represents one patient (*n* = 60); colors denote recovery groups.

**Table 3:** Spearman correlation matrix of all variables.

Variable	Precentral	Postcentral	SMA	Putamen	Pallidum	Thalamus	Baseline FMA-UE	Follow-up FMA-UE	ΔFMA-UE	MoCA	Age
Precentral		0.68	0.30	0.39	0.29	0.37	0.12	0.23	0.30	0.03	−0.14
Postcentral	0.68		0.10	0.54	0.36	0.40	0.04	0.15	0.30	0.03	−0.07
SMA	0.30	0.10		−0.07	−0.27	−0.12	0.12	0.12	0.03	0.04	−0.12
Putamen	0.39	0.54	−0.07		0.74	0.43	0.08	0.03	0.06	−0.04	0.03
Pallidum	0.29	0.36	−0.27	0.74		0.47	0.13	0.10	0.09	0.12	0.13
Thalamus	0.37	0.40	−0.12	0.43	0.47		0.05	0.01	0.07	−0.24	0.06
Baseline FMA-UE	0.12	0.04	0.12	0.08	0.13	0.05		0.67	−0.12	−0.04	−0.07
Follow-up FMA-UE	0.23	0.15	0.12	0.03	0.10	0.01	0.67		0.62	0.28	−0.18
ΔFMA-UE	0.30	0.30	0.03	0.06	0.09	0.07	−0.12	0.62		0.40	−0.13
MoCA	0.03	0.03	0.04	−0.04	0.12	−0.24	−0.04	0.28	0.40		−0.02
Age	−0.14	−0.07	−0.12	0.03	0.13	0.06	−0.07	−0.18	−0.13	−0.02	

Precentral = precentral gyrus AI; Postcentral = postcentral gyrus AI; SMA = supplementary motor area AI; Putamen = putamen AI; Pallidum = pallidum AI; Thalamus = thalamus AI. Precentral gyrus AI versus ΔFMA-UE: *r* = 0.299, *p* = 0.020; postcentral gyrus AI versus ΔFMA-UE: *r* = 0.299, *p* = 0.020. Bootstrap 95% confidence intervals for the correlations with ΔFMA-UE: precentral 0.02 to 0.54; postcentral 0.03 to 0.54; SMA −0.25 to 0.30; putamen −0.21 to 0.33; pallidum −0.18 to 0.36; thalamus −0.20 to 0.33. Benjamini–Hochberg FDR *q* values within the prespecified sensorimotor family (precentral, postcentral): both *q* = 0.020.

Because baseline MoCA scores differed between the two groups (*p* < 0.001) and MoCA score itself correlated with ΔFMA-UE (*r* = 0.404, *p* = 0.001), rank-transformed residual partial correlation analysis controlling for MoCA score was performed. The associations of both sensorimotor cortical AIs with ΔFMA-UE persisted (precentral gyrus: partial *r* = 0.305, *p* = 0.019; postcentral gyrus: partial *r* = 0.309, *p* = 0.017). After additionally controlling for baseline FMA-UE, the associations remained significant (precentral gyrus: partial *r* = 0.326, *p* = 0.013; postcentral gyrus: partial *r* = 0.314, *p* = 0.016), and they likewise persisted after further adjustment for lesion volume (precentral gyrus: partial *r* = 0.283, *p* = 0.033; postcentral gyrus: partial *r* = 0.266, *p* = 0.046).

To address regression to the mean and proportional-recovery effects, an ANCOVA sensitivity analysis with follow-up FMA-UE as the outcome and baseline FMA-UE as a covariate was performed. Precentral gyrus AI remained associated with the outcome (β = 0.13, 95% CI 0.01–0.25, *p* = 0.037), and the association persisted when MoCA score was additionally included (β = 0.11, 95% CI 0.01–0.22, *p* = 0.045). The corresponding association for postcentral gyrus AI was in the same direction but did not reach statistical significance (β = 0.09, 95% CI − 0.02 to 0.19, *p* = 0.128). Full partial-correlation and ANCOVA results are provided in Supplementary Table S1.

### 3.4 Multivariable linear regression analysis

A multivariable linear regression model combining the six regional CBF AIs with age and MoCA score was constructed for ΔFMA-UE. The combined model explained 29.7% of the variance in ΔFMA-UE (R^2^ = 0.297, adjusted R^2^ = 0.187), outperforming any single-region AI, and the overall model test was statistically significant (*F*(8,51) = 2.69, *p* = 0.015). Among individual predictors, only MoCA score was independently associated with ΔFMA-UE (B = 0.84, 95% CI 0.37–1.30, *p* < 0.001); the regional AI coefficients did not reach individual significance, consistent with shared variance among the correlated sensorimotor AIs. Detailed results of the univariable and multivariable regression analyses are provided in Table 4.

**Table 4:** Univariable and multivariable linear regression analyses.

Variable	Univariable B (95% CI)	*p*	Multivariable B (95% CI)	*p*	VIF
Precentral gyrus	0.126 (0.010, 0.243)	0.0376	0.130 (−0.076, 0.336)	0.2211	3.64
Postcentral gyrus	0.086 (−0.022, 0.193)	0.1245	0.007 (−0.179, 0.193)	0.9397	3.59
Supplementary motor area	0.040 (−0.176, 0.256)	0.7173	−0.087 (−0.307, 0.133)	0.4404	1.29
Putamen	0.044 (−0.106, 0.194)	0.5703	−0.004 (−0.219, 0.211)	0.9722	2.56
Pallidum	0.036 (−0.115, 0.187)	0.645	−0.066 (−0.318, 0.185)	0.6062	3.44
Thalamus	−0.029 (−0.233, 0.176)	0.7841	−0.018 (−0.276, 0.241)	0.8946	1.99
Age	—	—	−0.078 (−0.270, 0.114)	0.4314	1.10
MoCA score	—	—	0.838 (0.374, 1.302)	<0.001	1.27

Univariable models are independent linear regressions of each ROI AI on ΔFMA-UE (*n* = 60, df = 58). The multivariable model simultaneously included the six ROI AIs, age, and MoCA score (*n* = 60, df = 51): R^2^ = 0.297, adjusted R^2^ = 0.187, F(8,51) = 2.69, *p* = 0.015. A clinical-only model (baseline FMA-UE, age, and MoCA score) yielded R^2^ = 0.237; adding the precentral and postcentral gyrus AIs increased R^2^ to 0.286 (ΔR^2^ = 0.048, F change *p* = 0.170). A parsimonious four-predictor model (postcentral and precentral gyrus AI, MoCA score, baseline FMA-UE) yielded an apparent R^2^ of 0.272 (bootstrap optimism-corrected R^2^ = 0.162). VIF, variance inflation factor.

To evaluate the incremental value of the imaging measures, a clinical-only model (baseline FMA-UE, age, and MoCA score; R^2^ = 0.237) was compared with a model adding the precentral and postcentral gyrus AIs (R^2^ = 0.286; ΔR^2^ = 0.048, F change *p* = 0.170); this increment did not reach statistical significance at the current sample size and is reported transparently. A parsimonious four-predictor model (postcentral and precentral gyrus AI, MoCA score, baseline FMA-UE; events-per-variable ratio = 15) yielded an apparent R^2^ of 0.272; bootstrap internal validation (1,000 resamples) gave an optimism-corrected R^2^ of 0.162. The eight-predictor model is reported as an apparent in-sample estimate only.

## 4 Discussion

### 4.1 Principal findings

In 60 patients with AIS whose baseline motor function did not differ significantly between groups (*p* > 0.05), this study evaluated the associations between acute-phase (< 72 h) CBF asymmetry indices (AIs) in six bilateral motor-related regions and 30-day upper-limb motor recovery. Three principal findings emerged. First, continuous-variable analysis showed moderate positive correlations of both precentral gyrus AI (*r* = 0.299) and postcentral gyrus AI (*r* = 0.299) with ΔFMA-UE, whereas correlations for the SMA, putamen, pallidum, and thalamus AIs were negligible (|*r*| < 0.09); both sensorimotor cortical associations survived FDR control within the prespecified testing family. Second, the associations were robust to adjustment for the two variables that differed between groups: they persisted after controlling for MoCA score, baseline FMA-UE, and lesion volume (partial r up to 0.326), and precentral gyrus AI remained associated with follow-up motor function in an ANCOVA framework that accounts for regression to the mean (*p* = 0.037). Third, a multivariable model combining the six regional AIs with age and MoCA score explained 29.7% of the variance in ΔFMA-UE (adjusted R^2^ = 0.187) and reached overall statistical significance (*p* = 0.015), although the increment over clinical variables alone did not reach significance (ΔR^2^ = 0.048, *p* = 0.170). Group comparisons further showed smaller absolute AIs in the sensorimotor cortex of the good recovery group, a pattern consistent with more symmetric bilateral perfusion; because the AI is a relative measure, it does not by itself demonstrate preservation of absolute ipsilesional CBF. These results suggest that acute-phase perfusion symmetry of the sensorimotor cortex, rather than unilateral perfusion level alone, may carry information relevant to early motor recovery.

### 4.2 Potential role of sensorimotor cortex perfusion asymmetry: a sensorimotor integration perspective

A notable finding of this study was that the regional AIs most strongly correlated with ΔFMA-UE were those of the sensorimotor cortex—both the primary motor cortex (precentral gyrus) and the primary somatosensory cortex (postcentral gyrus)—with nearly identical correlation magnitudes (both *r* ≈ 0.30). This finding is consistent with the recent theoretical emphasis on restoring the sensory component to models of sensorimotor control (Edwards et al., 2019). Somatosensory feedback is a necessary input for motor plan revision and motor learning. A systematic review showed that combined light-touch and proprioceptive deficits are significantly associated with upper-limb motor recovery and participation in daily activities (Meyer et al., 2014). In a large-sample imaging–behavior mapping study, Kessner et al. (2019) showed a clear cortical localization of somatosensory deficits after ischemic stroke. Consistently, Zhou et al. (2021) demonstrated impaired cortico-cortical connectivity underlying fine tactile sensation deficits after stroke, underscoring the sensory side of sensorimotor disruption. Most importantly, a longitudinal study by Zandvliet et al. (2020) found that early recovery of somatosensory function may be a conditional prerequisite for upper-limb motor recovery, with almost no significant motor improvement among patients whose sensory function did not recover. The perfusion data from the present study add a hemodynamic dimension to this behavioral evidence. Patients with more symmetric sensorimotor cortex perfusion (AI closer to 0) achieved greater motor improvement. This finding suggests, but does not establish, that the perfusion reserve of the sensorimotor cortex may support recovery by maintaining sensorimotor integration capacity.

Viewed in the context of previous evidence, these findings support a provisional serial model: local perfusion is positively correlated with microstructural integrity within days after stroke (Kiely et al., 2023), and microstructural integrity of the ipsilesional motor pathway explains a large proportion of the variance in subsequent motor scores (Kim et al., 2025; Zhan et al., 2023). One plausible physiological pathway is that preserved perfusion supports tissue viability, maintains sensory feedback loops, and enables motor learning, thereby linking the acute-phase perfusion phenotype to functional recovery. The anatomical specificity of our findings is also consistent with the cortical nodes required for motor execution and proprioceptive feedback: the precentral gyrus (primary motor cortex, BA 4), SMA (medial BA 6), and postcentral gyrus (somatosensory cortex, BA 1–3) are key neural structures involved in voluntary motor control and sensory integration (Abougalala, 2024; Li et al., 2024; Unger et al., 2023). This anatomical correspondence suggests that perfusion-based stratification targets functionally relevant circuits rather than nonspecific compensatory recruitment. Nevertheless, because this study used a correlational design, the proposed causal pathway remains a hypothesis that requires validation in longitudinal interventional studies.

An alternative explanation that warrants consideration is confounding by baseline cognition: the good recovery group had higher MoCA scores (26.30 ± 2.29 versus 23.40 ± 2.94, *p* < 0.001), and MoCA score itself correlated with ΔFMA-UE (*r* = 0.404, *p* = 0.001). We considered this issue in two ways. First, statistically, partial correlation analysis controlling for MoCA showed that the associations of both sensorimotor cortical AIs with ΔFMA-UE persisted (partial *r* = 0.305 and 0.309, both *p* < 0.02), and they further persisted after controlling for baseline FMA-UE and lesion volume. Second, anatomically, the postcentral gyrus is a primary somatosensory region and is not generally regarded as a principal substrate of the core cognitive domains assessed by the MoCA (executive function, memory, visuospatial function, and language); therefore, its association with motor recovery is unlikely to be explained entirely by cognitive reserve. The persistence of both sensorimotor cortical associations after controlling for MoCA provides additional, although not definitive, support for sensorimotor integration rather than general cognitive status as the principal explanation for these findings. Nevertheless, the MoCA is only a screening instrument, and residual confounding may remain (see Section 4.6).

### 4.3 Interhemispheric perfusion imbalance and the double-edged nature of contralateral compensation

Group comparisons showed larger absolute AIs in the sensorimotor cortex of the poor recovery group (for the precentral gyrus, Cohen’s d = 0.41, a small-to-medium effect size), although the reported between-group differences were not statistically significant. This pattern is consistent with relatively lower ipsilesional or relatively higher contralesional perfusion and can be interpreted within the theoretical framework of interhemispheric interactions. The classic interhemispheric competition model proposes that the contralesional hemisphere exerts abnormally enhanced transcallosal inhibition on the ipsilesional hemisphere, thereby hindering recovery (Bencivenga et al., 2023; Keser et al., 2022). The bimodal balance–recovery model proposed by Di Pino et al. further suggests that the role of contralesional activity depends on the degree of structural reserve: when reserve is relatively preserved, contralesional overactivity is mostly maladaptive, whereas when reserve is depleted, contralateral compensation may become necessary to maintain residual function (Di Pino et al., 2014). The patients enrolled in this study had mild-to-moderate deficits (NIHSS 5–15, baseline FMA-UE approximately 25) and may represent a population with relatively preserved structural reserve, although structural reserve was not measured directly. Recovery in such patients may be driven mainly by the ipsilesional network; therefore, the more pronounced perfusion imbalance in the poor recovery group may reflect an inefficient or maladaptive pattern of blood flow distribution. This interpretation is compatible with interventional evidence that mirror therapy facilitates stroke recovery by recruiting ipsilateral motor pathways (Zhang et al., 2024).

Thamm et al. found in a multicenter cohort dominated by anterior-circulation large-vessel occlusion that higher absolute CBF in the contralesional hemisphere was associated with better 90-day functional outcomes (Thamm et al., 2019). This finding appears inconsistent with the present finding that more symmetric bilateral perfusion was associated with better recovery. However, the populations and metrics of the two studies differ. The iCAS cohort mainly comprised patients with large-vessel occlusion who were candidates for endovascular treatment, in whom absolute contralesional CBF may reflect collateral recruitment capacity and systemic perfusion status. In contrast, the present study excluded patients who received reperfusion therapy and used a bilateral relative ratio (AI) to characterize the balance of blood flow distribution within the motor network. The findings may therefore be complementary rather than contradictory: absolute perfusion level and relative symmetry may represent two dimensions that carry different physiological information. Future studies should examine both in the same cohort to clarify their interaction.

### 4.4 Value and limitations of a combined multi-region assessment

A single-region AI had limited explanatory power for ΔFMA-UE (R^2^ ≤ 0.072), whereas combining the six regional AIs with clinical variables increased R^2^ to 0.297, and the combined model reached overall statistical significance (*F*(8,51) = 2.69, *p* = 0.015). This result is consistent with the view that motor recovery is related to network-level perfusion status rather than perfusion in a single region and with connectomic evidence of post-stroke brain network reorganization (Grefkes and Fink, 2011). However, several caveats must be emphasized. First, the incremental value over a clinical-only model did not reach statistical significance (ΔR^2^ = 0.048, F change *p* = 0.170); the imaging measures therefore add explanatory value that is correlated with, rather than clearly independent of, clinical variables at this sample size. Second, with eight predictors and 60 patients, the adjusted R^2^ decreased to 0.187, and bootstrap internal validation of a parsimonious four-predictor model reduced the apparent R^2^ from 0.272 to an optimism-corrected 0.162, indicating substantial overfitting risk. The reported coefficients of determination should be regarded as apparent in-sample estimates. Compared with sequentially validated prediction frameworks such as PREP and PREP2 (Stinear et al., 2012; Stinear et al., 2017), the combined model at this stage should be regarded only as a hypothesis-generating tool. Its coefficient estimates and variable composition require recalibration and validation in external cohorts. In addition, the proportional recovery phenomenon suggests that initial deficit severity explains a substantial proportion of recovery variance in unstratified populations (Byblow et al., 2015). The present study partially addressed this confounding through the primary continuous-variable analysis, ANCOVA with baseline motor function as a covariate, and a group design in which baseline motor function was comparable between groups, but future studies should incorporate the initial deficit × time interaction into the model to distinguish spontaneous recovery from perfusion-related differences in recovery.

The present findings should also be positioned cautiously within the broader evidence base for perfusion imaging. The American Academy of Neurology (AAN) evidence-based guideline rated diffusion-weighted imaging (DWI) lesion measures as Level B evidence for the diagnosis and prognosis of acute stroke, whereas perfusion-weighted imaging was rated Level C, meaning possibly useful but supported by insufficient evidence (Schellinger et al., 2010). To date, validated imaging markers of motor prognosis remain predominantly structural, particularly quantitative measures of corticospinal tract integrity (Hayward et al., 2022; Puig et al., 2017). Therefore, the perfusion asymmetry findings should be viewed as a complement to, rather than a replacement for, the existing framework of structural markers. Their incremental explanatory value should be tested directly in cohorts in which structural and perfusion imaging are acquired concurrently.

### 4.5 Prospects for clinical translation

These findings suggest a potential workflow for evaluation in acute stroke units. ASL could be added to the routine 72-h MRI [acquisition time < 5 min, no contrast agent (Alsop et al., 2015; Iutaka et al., 2023)], and motor-related regional AIs could be calculated using the standardized AAL atlas to provide exploratory perfusion stratification information before rehabilitation begins. Neuroradiologists could interpret these measures using standardized ROI templates. If validated, this information could have two potential uses. First, it may help identify patients with a favorable perfusion phenotype (sensorimotor cortex AI closer to zero, i.e., more symmetric bilateral perfusion) as candidates for trials of high-intensity, ipsilesionally focused training such as constraint-induced movement therapy (CIMT). The EXCITE randomized controlled trial and subsequent systematic reviews have shown benefits of such intensive interventions in patients with some residual motor function (Kwakkel et al., 2015; Wolf et al., 2006), and perfusion stratification may help address the unresolved question of who is most likely to benefit. Second, in patients with marked perfusion imbalance, the addition of sensory retraining to standard motor training or the selection of noninvasive brain stimulation or brain-computer interface-based motor training strategies (Pichiorri et al., 2023) according to the bimodal model could be investigated as approaches to modulate interhemispheric balance (Di Pino et al., 2014; Edwards et al., 2019). Consistent with the individualized stratification principles advocated by the American Heart Association/American Stroke Association stroke rehabilitation guidelines (Winstein et al., 2016), ASL-based perfusion phenotyping could ultimately complement clinical scales and corticospinal tract integrity measures in a multimodal stratification framework (Boyd et al., 2017; Feng et al., 2015; Stinear et al., 2012).

### 4.6 Limitations and future directions

This study has several limitations. First, although the cohort was enlarged to 60 patients, the sample size remains limited and the study was conducted at a single center. Patients who received intravenous thrombolysis or mechanical thrombectomy were not included, which may have introduced selection bias; generalizability therefore requires validation in multicenter studies that include standard reperfusion populations. For the primary analysis—the correlations between sensorimotor cortical AIs and ΔFMA-UE—the study provided 80% power only for correlations of *r* ≥ 0.35; the observed associations (*r* = 0.299, *p* = 0.020) were therefore detected with limited power (approximately 65%) and should be regarded as exploratory. The observed between-group effects (largest Cohen’s d = 0.41) were small to medium, and precision for smaller effects in the secondary and exploratory analyses is limited; their null findings should be interpreted with caution. Second, the 30-day follow-up captured only early recovery and may have missed later improvement. Given the time-window characteristics of proportional recovery (Byblow et al., 2015), extending follow-up to 90 days and 6 months is particularly important. Third, baseline MoCA scores differed between groups. Although the sensorimotor cortical associations persisted after controlling for MoCA in partial correlation analyses, the MoCA is a screening instrument and provides only limited control for residual confounding from unmeasured dimensions such as attention and executive function. Fourth, lesion volume was larger in the poor recovery group (*p* = 0.005) and correlated negatively with ΔFMA-UE (*r* = −0.412); although the AI associations persisted after adjustment for lesion volume (partial *r* = 0.283 and 0.266 for the precentral and postcentral gyri), and the AI is a ratio-based measure that is relatively insensitive to absolute lesion size, residual confounding by lesion burden cannot be excluded. Fifth, because ΔFMA-UE change scores can be affected by regression to the mean and proportional-recovery effects, the key associations were re-examined with ANCOVA using follow-up FMA-UE as the outcome and baseline FMA-UE as a covariate; the precentral gyrus association persisted (*p* = 0.037), whereas the postcentral gyrus association was attenuated (*p* = 0.128), and this asymmetry should be considered when interpreting the regional specificity of the findings. Sixth, the descriptive dichotomization at ΔFMA-UE ≥ 10 / ≤ 4 was chosen to represent clinically unambiguous recovery states outside the MCID range; no enrolled patient fell between the two thresholds, but the grouping remains *post hoc* and was used for description only, with all primary inferences based on continuous-variable analyses. The absence of patients with ΔFMA-UE between 5 and 9 points deserves comment: rather than reflecting selection, this gap is compatible with the bimodal recovery pattern described in the proportional-recovery literature, in which patients cluster into those who recover a large proportion of their initial deficit and those who recover little, with relatively few patients in the intermediate band (Byblow et al., 2015); because enrollment was consecutive and the dichotomization excluded no one, the gap cannot have resulted from our grouping procedure, although we cannot exclude chance variation in a cohort of this size. Seventh, ASL was acquired with a single PLD of 2,050 ms without arterial transit time correction, and vascular imaging (MRA/CTA) was not performed in the study protocol. Delayed arterial transit—potentially present in patients with arterial stenosis or collateral-dependent flow—can lead to underestimation of CBF in the affected territory with single-PLD acquisition. Several features mitigate, but do not eliminate, this concern: the PLD of 2,050 ms follows the consensus recommendation for populations with expected delayed transit (Alsop et al., 2015); the cohort comprised mild-to-moderate, non-reperfused patients, among whom proximal large-vessel occlusion is uncommon; and the AI associations persisted after adjustment for lesion volume, a partial proxy of stroke severity. Any residual random transit-related noise would be expected to attenuate rather than inflate the observed correlations, although a systematic influence cannot be excluded; multi-PLD ASL with transit-time mapping should be used in future validation studies.

Future studies should: (i) externally validate the perfusion phenotype and combined model in larger multicenter cohorts; (ii) extend follow-up to 90 days and 6 months to characterize the full recovery trajectory; (iii) use longitudinal ASL designs to examine the dynamic evolution of perfusion after the acute phase and its temporal relationship with recovery; and (iv) conduct prospective interventional trials stratified by perfusion phenotype to test whether intensive rehabilitation guided by this stratification improves clinical outcomes.

## 5 Conclusion

Early (<72 h) ASL-derived cerebral blood flow asymmetry patterns were associated with 30-day upper-limb motor recovery in patients with AIS. Among patients with comparable baseline motor function, those with more symmetric bilateral perfusion in the sensorimotor cortex (AI closer to zero) tended to achieve greater FMA-UE recovery, and these associations were robust to adjustment for cognitive status, baseline motor function, and lesion volume. A model combining multi-region CBF AIs with clinical variables explained approximately 30% of the variance in motor recovery (adjusted R^2^, approximately 19%) and reached overall statistical significance (*F*(8,51) = 2.69, *p* = 0.015); however, its incremental value over clinical variables alone was not statistically significant, and the model should currently be regarded as a hypothesis-generating tool rather than a validated predictor. The findings provide exploratory support for noninvasive assessment of recovery potential during the acute phase. Future studies should validate the clinical utility of this approach in larger multicenter cohorts and extend follow-up to evaluate long-term recovery trajectories.

## Data Availability

The raw data supporting the conclusions of this article will be made available by the authors, without undue reservation.
