# Supplementary material for: Cerebral blood flow asymmetry in motor-related regions is associated with 30-day upper-limb motor recovery after acute ischemic stroke
Source: Front Neurosci. 2026 Oct 30;20:1955245. doi: 10.3389/fnins.2026.1955245 (PMC13759772; doi:10.3389/fnins.2026.1955245)
Supplement: Supplementary file 1 [file Table_1.DOCX]

**Supplementary Material**

Cerebral Blood Flow Asymmetry in Motor-Related Regions Is Associated With 30-Day Upper-Limb Motor Recovery After Acute Ischemic Stroke

*Contents: Supplementary Table S1 (partial correlation and ANCOVA sensitivity analyses); Supplementary Table S2 (sensitivity analysis with an alternative ΔFMA-UE cut-off).*

**Supplementary Table S1 Partial correlation and ANCOVA sensitivity analyses**

| **ROI** | **Partial r (MoCA)** | **P** | **Partial r (MoCA + baseline FMA-UE)** | **P** | **ANCOVA β (95% CI)** | **P** | **ANCOVA + MoCA, P** |
| --- | --- | --- | --- | --- | --- | --- | --- |
| Postcentral gyrus | 0.309 | 0.017 | 0.314 | 0.016 | 0.09 (-0.02, 0.19) | 0.128 | 0.110 |
| Precentral gyrus | 0.305 | 0.019 | 0.326 | 0.013 | 0.13 (0.01, 0.25) | 0.037 | 0.044 |

Note: Partial correlations are Pearson correlations of rank-transformed residuals. ANCOVA models used follow-up FMA-UE as the outcome and baseline FMA-UE as a covariate, with and without additional adjustment for MoCA score. After further adjustment for lesion volume in the rank-residual partial correlation framework (controlling MoCA + baseline FMA-UE + lesion volume), the associations persisted (precentral gyrus: partial r = 0.283, P = 0.033; postcentral gyrus: partial r = 0.266, P = 0.046).

**Supplementary Table S2 Sensitivity analysis with an alternative ΔFMA-UE cut-off (≥ 5)**

| **ROI** | **Good recovery mean AI** | **Poor recovery mean AI** | **t** | **P** |
| --- | --- | --- | --- | --- |
| Precentral gyrus | -4.70 | -9.36 | 1.600 | 0.116 |
| Postcentral gyrus | -4.11 | -7.29 | 0.980 | 0.333 |
| Supplementary motor area | -2.60 | -3.27 | 0.402 | 0.689 |
| Putamen | -4.64 | -4.97 | 0.141 | 0.889 |
| Pallidum | -2.89 | -3.16 | 0.115 | 0.909 |
| Thalamus | -4.98 | -4.06 | -0.522 | 0.603 |

Note: No enrolled patient had a ΔFMA-UE between 5 and 9 points; the ≥ 5 cut-off therefore produced a grouping identical to the primary ≥ 10 / ≤ 4 grouping, and the results are identical by construction. This confirms that the descriptive group comparison is insensitive to the choice of cut-off within the 5–10 range.
